# The Role of Graphene Oxide Nanocarriers in Treating Gliomas

**DOI:** 10.3389/fonc.2022.736177

**Published:** 2022-01-28

**Authors:** Bin Wang, Hanfei Guo, Haiyang Xu, Yong Chen, Gang Zhao, Hongquan Yu

**Affiliations:** ^1^Department of Neurosurgery, The First Hospital of Jilin University, Changchun, China; ^2^Cancer Center, The First Hospital of Jilin University, Changchun, China

**Keywords:** antitumor, cell cycle, graphene oxide, immunotherapy, invasion, metabolism, migration, phototherapy

## Abstract

Gliomas are the most common primary malignant tumors of the central nervous system, and their conventional treatment involves maximal safe surgical resection combined with radiotherapy and temozolomide chemotherapy; however, this treatment does not meet the requirements of patients in terms of survival and quality of life. Graphene oxide (GO) has excellent physical and chemical properties and plays an important role in the treatment of gliomas mainly through four applications, viz. direct killing, drug delivery, immunotherapy, and phototherapy. This article reviews research on GO nanocarriers in the treatment of gliomas in recent years and also highlights new ideas for the treatment of these tumors.

## 1 Introduction

Glioma, originating from neuroectodermal cells, is the most common primary malignant tumor of the central nervous system and is aggressive and lethal. Gliomas account for 75% of intracranial primary malignant tumors and have a high morbidity and mortality (1, 2). Glioblastoma multiforme (GBM) is the most common malignant type, accounting for approximately 57% of all gliomas (3). At present, the standard treatment for GBM is simultaneous maximal safety surgical resection and chemoradiotherapy with subsequent adjuvant chemotherapy for 6 months (4). Owing to the invasive and temozolomide–resistant nature of GBM, the 1-year survival rate of patients with the disease is approximately 41.4% whereas the 5-year survival rate is less than 5% (5). In recent years, much progress has been made in the diagnosis and treatment of gliomas (6, 7). The proposal of molecular pathology allows for a more precise diagnosis, which will enable more effective therapeutic approaches (8). The optimization of traditional therapy includes easing the intra-operative delineation and improving spatial and time radio exposure (9, 10). Tumor treating fields (TTF) is a newly approved therapeutic approach in high-grade gliomas patients, which is both effective and safe (11). In addition, new therapies such as molecular targeting and immunotherapy have also accumulated more and more experience, but few achievements can change clinical practice protocols or outcomes (12–14). Nanocarriers are pharmaceutically–relevant colloidal systems with sizes in the range of 1–1000 nm. These colloidal systems are capable of treating tumors through direct action or their ability to as drug delivery systems (15, 16). The applicability of these nanocarriers in tumor targeting and treatment is accomplished through the following: 1) loading anti-tumor drugs through their ability to act as drug delivery systems, 2) functional modification of the drug-loaded nanoparticles by adding targeting ligands, and 3) using specific physics, chemistry, or biological methods to release the drug in the appropriate amount to exert the desired therapeutic effect (17, 18). Graphene oxide (GO) nanocarriers have the characteristics of easy modification and good dispersibility and are thus postulated to become a new nanocarrier-based therapeutic option for the management of gliomas. Consequently, research on GO and GO nanocarriers has gradually become a hot topic in recent years (19).

## 2 Overview of Graphene Oxide Nanocarriers

### 2.1 Graphene Oxide

Graphene is a 2-dimensional sheet of sp^2^-hybridized carbon with a honeycomb-like structure (20). Graphene has a relatively complete structure, high stability, and weak interaction with other media, which is not conducive to its use as a drug carrier. GO and reduced GO (rGO) are derivatives of graphene. The surfaces of GO and rGO contain a large number of oxygen-containing functional groups such as carboxyl, epoxy, and hydroxyl groups, which can be chemically modified as active sites (21). The existence of a large number of oxygen-containing groups leads to good water solubility and dispersibility of GO and rGO (22). The skeleton of GO and rGO is an aromatic ring with a large specific surface area and abundant functional groups, which can increase drug loading *via* π–π interactions and bind biological macromolecules, such as proteins, nucleotide acid fragments, and aptamers to facilitate functional modification and target recognition (22, 23) (**Table 1**).

**Table 1:** A brief summary of functional modifications of GO.

Graphene composite	Modification material	Observations	Ref.
GO	Arginine-Glycine-Aspartate (RGD)	Improving tumor-targeting efficiency	(24)
GO	Polyethylene glycol (PEG)	Improving biocompatibility and drug delivery capacity of GO	(25)
GO	Transferrin (Tf)	Improving tumor-targeting efficiency	(26)
GO	Monoclonal antibody (mAb)	Improving tumor-targeting efficiency	(27)
GO	Carboxymethyl chitosan (CMC)	Improving biocompatibility and drug delivery capacity of GO	(28)
GO	Folic acid (FA)	Improving tumor-targeting efficiency	(29, 30)
GO	Lactosylated chitosan oligosaccharide (LCO)	Improving tumor-targeting efficiency and anti-tumor genes delivery capacity of GO	(31)
GO	Polyethylenimine (PEI)	Improving biocompatibility and drug delivery capacity of GO	(32)
GO	β-Cyclodextrin (β-CD)	Improving photothermal efficiency	(33)
GO	Iron oxide nanoparticle (IONP)	Improving photothermal efficiency and MRI sensitivity	(34)
GO	Chlorotoxin (CTX)	Improving drug delivery capacity of GO	(35)
GO	Chitosan (CS)	Improving biocompatibility and drug delivery capacity of GO	(36)
GO	Poly N-vinyl caprolactam (PVCL)	Improving biocompatibility and drug delivery capacity of GO	(37)
GO	Hyaluronic acid (HA)	Improving tumor-targeting efficiency and drug delivery capacity of GO	(38)
GO	Pluronic F127 (PF127)	Improving biocompatibility of GO	(39, 40)
GO	Glycyrrhetinic acid (GA)	Improving tumor-targeting efficiency	(41)
GO	Polyetheramine (PEA)	Improving biocompatibility and drug delivery capacity of GO	(42)
GO	Poly (acrylic acid) (PAA)	Improving biocompatibility of GO	(43)

### 2.2 Graphene Oxide Nanocarriers

The tumor cells are growing disorderly and dense, so conventional drug delivery systems work only on the surface of the tumor, making it difficult to penetrate deep into the tumor tissue (44). Molecules and nanomaterials with sizes ranging between 10 and 100 nm can be retained in tumors because of the abnormal blood vessels and the defective lymphatic circulation in tumor tissues. This is known as the enhanced permeability and retention effect (EPR) (45–47). EPR provides an option to achieve spontaneous accumulation of GO for the passive delivery of anti-tumor drugs to tumors. However, EPR provides less than a 2-fold concentration increase in tumor tissues compared with non-target tissues and organs, and sometimes the efficiency of EPR is too low to reach the drug concentrations sufficient for curing cancer (48). There is difficulty in translating the EPR effect from bench to bedside because of intra- and inter-tumoral heterogeneity, heterogeneity in nanoparticle accumulation in the different tumors, and EPR effects differences between rodent tumors and human tumors (49–52). The GO nanocarrier system has more advantages than the traditional anti–tumor drug delivery system, like liposomes. These include ① good blood compatibility and optimal dispersibility in the liquid environment of the human body (53); ② large specific surface area facilitating multi–functional modification by biomolecules and small molecules, such as proteins and single–stranded DNA bases (54); ③ single atomic layer of thickness, and an ultra–high drug loading capacity compared with other nanomaterials (55); ④ sustained release and prolonged drug half-life (56); ⑤ relatively good biological safety and acceptable toxicity (57–61). Various modification methods have been employed to improve the solubility, stability, and cytotoxicity of GO, while there is still a need to further evaluate the biocompatibility and toxicity of GO nanocarriers into the human body (57, 62).

### 2.3 Functional Modification of Graphene Oxide Nanocarriers

Graphene is too stable to react with other materials, which limits its application as a drug nanocarrier; however, GO can easily be functionally modified (63, 64). Functional modification of the structure and properties of GO is an effective way to improve the utilization of graphene carriers. At present, functional modification methods for GO are mainly divided into two types: covalent and non-covalent modifications. Covalent modification refers to the use of chemical reactions to modify the oxygen-containing functional groups on the surface of GO. The application of non-covalent bond modification is ingenious. It uses π–π conjugation, ionic bonds, and hydrogen bonds to modify GO. Functional modification can provide new properties to GO and thus enhance the dissolution of GO in polar solvents to improve its dispersibility (65).

#### 2.3.1 Covalent Modification of Graphene Oxide Nanocarriers

The surface of GO contains a large number of active oxygen groups, such as hydroxyl, carboxyl, and carbonyl groups, which can chemically react with other groups through covalent bonds to optimize its performance by forming interactions such as ester and amide bonds. Marcelo et al. used D–mannose to covalently modify GO (man–GO) using mannosylated ethylenediamine, which was found to improve the RBC toxicity and protein corona formation of GO (66). Covalent modification of GO is important in therapeutic pathways. Ouyang et al. covalently modified GO with arginine–glycine–aspartic acid (RGD) and silicon phthalocyanine (SiPc), which is a fluorescence imaging-guided photothermal and photodynamic therapy for cancer (67). Chen et al. further bound GO to anti-HER2 antibodies loaded with dual-drug DOX and 9-aminoacridine, which increased cytotoxicity against cancer cells (27). Both RGD and anti-HER2 antibodies are one of the motifs helping improve active tumor-targeting properties. FA also acts as a molecular recognition motif for folate-receptor-positive cancer cells (68). Zhuang et al. covalently linked pGO-FA to hydrophobic paclitaxel (PTX) to overcome its water-insoluble, which exhibited a higher efficiency in killing A2780 cells (69). Xu et al. covalently modified GO with PEG, forming a high potential drug delivery system in cancer therapy. PTX was linked to GO-PEG, exhibiting great water solubility and cancer-killing efficiency (70, 71). More et al. covalently linked iron oxide nanoparticles (IONPs) with GO forming GOIOI, which showed great potential in cancer metastasis (72). The modification of the GO surface *via* a covalent route is strong and not susceptible to the inconsistent external environment, which makes the GO nanocarriers stable with biological systems *in vitro* or *in vivo*.

#### 2.3.2 Non–Covalent Modification of Graphene Oxide Nanocarriers

The surface of GO has many charges, which can be functionally modified by DNA, some small molecule drugs, or small molecules through non-covalent bonds, such as π–π conjugation, ionic bonds, and hydrogen bonds, making GO an ideal nanocarrier for drug delivery. Pan et al. combined 5-FU with Tau-GO through a non-covalent bonding mechanism to form 5-FU–Tau-GO, which was reported to continuously release 5-FU in the acidic environment of the tumor, thus prolonging the duration of action of 5-FU more effectively than 5-FU alone (73). Wang et al. non-covalently modified GO with PF127 and doxorubicin (DOX), which exhibited a high loading capacity and better biocompatibility (39). Li et al. established non-covalent interaction between trastuzumab (TRA) and GO, enhancing HER2-binding activity to kill osteosarcoma (74). Lin et al. found that noncovalently combined GO with hypocrellin A (HA) can be excited by light irradiation and cause tumor cell death *in vitro* (75). Li et al. developed an anticancer drug delivery system by the decoration of GOIOI with doxorubicin and non-covalent PEGylation, which combined chemotherapy with phototherapy (34). Wang et al. noncovalently loaded DOX onto GO-chlorotoxin (CTX), and DOX showed in pH-dependent sustained-release manner (35). However, noncovalently modified GO may load fewer aromatic drugs than covalently modified GO.

### 2.4 Biocompatibility of Graphene Oxide Nanocarriers

The potential toxic effect of GO on living cells and organs limits its use in cancer therapy. Clinical use of GO requires extensive studies on its potential short- and long-term toxicity *in vivo* and *in vitro*, mainly through cell experiments and animal models to assess its biosafety. GO is much less toxic than graphene since the surface of GO is rich in a large number of carboxyl, hydroxyl, epoxy, and other active functional groups, which induces good water-solubility and biocompatibility of GO (76, 77). Lu et al. reported that the relative survival rate of cells was nearly 100% even with 100mg/L of Go, indicating good biosafety levels of GO in the cell type tested (78). Several studies have shown that cytotoxicity of GO can decrease after modification with biocompatible substances such as PEG, chitosan (79–81). Kai et al. functionalized graphene with PEG, and PEGylated graphene showed high tumor uptake in several xenograft tumor mouse models, which denoted no obvious side effect (82). Zhuang et al. functionalized GO with PEG, which was stable in various biological solutions and showed no obvious increase of apoptosis even at very high concentrations in HCT-116 cells (83). Sowmya et al. reported that few-layer graphene didn’t elicit cell death on primary macrophages (84). Marco et al. investigated the effects of large and small GO on human peripheral blood mononuclear cells (PBMCs), and there are no significant differences in human PBMCs viability by the exposure to both GO types (85). Sayan et al. reported that GO-PEG-DSPE was meaningfully toxic to U251 but not toxic to normal cells, and the differences could be due to the specific receptors on the surface of glioma cells (86). Portioli et al. directly injected GO in mice brains and assessed the induction of acute neuroinflammatory and neurotoxic effects locally and distantly from the injection site one week post-administration (87). Compared to the liposome group, none of the GO group’s mice induced either neuroinflammatory or neurotoxic effects, and only moderate activation of proinflammatory makers was induced at the molecular level in the GO group. However, GO nanocarriers have a toxicity hazard depending on the route of administration, the dose to be administered, the method of synthesis of GO, and its physicochemical properties, which needs an in-depth, thorough evaluation in different animal models (57). Cao et al. reported that GO could be taken into human umbilical vein endothelial cells (HUVEC) leading to cytotoxic effects by activation of endoplasmic reticulum (ER) stress and pyroptosis genes (58). Inhalation, ingestion, and dermal are the major routes of entry of GO, and intravenous GO is distributed mainly in the lungs, liver, and spleen (57). GO and rGO exhibit potential toxicity in non-target organs in laboratory mammals, including liver, lung, spleen, kidney, and reproductive organs, which need further investigation (62).

## 3 Application of Graphene Oxide Nanocarriers in the Treatment of Glioma

### 3.1 Direct Killing of Graphene Oxide

In their traditional applications and understanding, GO and rGO are known as excellent drug nanocarriers. Slawomir et al. have shown that both GO and rGO have a direct killing effect on gliomas and that rGO has a stronger killing effect through inducing apoptosis of glioma cells *in vitro* and *in vivo* (88, 89). GO has anti-proliferative and anti-migratory effects in gliomas. Maciej et al. postulated that the underlying mechanism of GO is related to its effect on the expression of oxidative phosphorylation genes in glioblastoma U87 cell line (90). In addition, Mateusz et al. showed that GO can decrease migration and invasiveness of gliomas by impairing extracellular adhesion and regulating adhesion-dependent pathways, such as EGFR/AKT/mTOR and β-catenin in two glioblastoma cell lines, U87 and U118 (91). Tian et al. showed that GO can retard migration by direct disruption of actin filaments *in vitro*, which is a novel application of GO (92). Xu et al. found that GO may damage the integrity and function of the cell membrane *in vitro* and *in vivo* by downregulating cytoskeleton-related genes, such as Actg2, Tubb2a, and Neb (43). In another study, Mateusz et al. showed that GO can inhibit the angiogenesis and tumor microenvironment of U87 (p53 wild) but not U118 (p53 mutant) *via* the NF-κB pathway, indicating that the regulation of gliomas by GO is related to the status of p53 (93). Glioblastoma stem-like cells (GSCs) contribute to the self-renewal and rapid growth of glioblastoma and likely drive the onset of tumor growth, therapeutic resistance, and tumor recurrence. Marco et al. reported that GO could induce GSCs differentiation *in vitro* by inhibiting several signal transduction pathways (WNT/β-catenin, Notch, and JAK-STAT) (94). Wang et al. found that GO induces cell cycle arrest and differentiation of GSCs by epigenetic regulation of GSCs, thereby inhibiting the growth of gliomas *in vitro* and *in vivo* (95). GO exerts an anti-glioma effect by anti-proliferation, anti-migration, anti-angiogenesis, and anti-GSC actions; nonetheless, the exact mechanisms still require further research. Jaroslaw et al. revealed that rGO can promote the apoptosis of gliomas through caspase- and mitochondrion-dependent apoptotic pathways and has a direct killing effect on gliomas in glioblastoma U87 cell line (96). In another study, Jaroslaw found that rGO could reduce the expression of voltage-dependent ion channel genes and extracellular receptors in U87 cells, and induce the damage of cell membrane and the changes the of cell membrane potential (97). However, rGO was shown not to specifically distribute to gliomas and rather agglomerated after reaching the gliomas, thus affecting its anti-tumor function. Ewa et al. used arginine (Arg) to modify rGO to enhance its specific distribution around gliomas and avoid rGO agglomeration *in vivo* with GBM tumors cultured on chicken embryo chorioallantoic membranes. Following the modification, rGO–Arg was found to block MDM2 expression and upregulate NQO1 expression, thereby optimizing its effect on gliomas (98). However, further *in vivo* experiments are still needed.

### 3.2 Graphene Oxide Nanocarriers as Drug Delivery Systems

GO and rGO are the most commonly described drug delivery nanocarriers having abundant oxygen-containing groups. The ideal target agent carrier should have four characteristics: targeting desired cells, controlled drug release, non-toxic to normal cells, and biodegradable. For intracranial tumors, one of the common obstacles is delivery of chemotherapy drugs to the tumor site owing to the presence of the blood–brain barrier (BBB) (99). GO can penetrate the BBB, and increase drug accumulation through EPR in gliomas (46, 47, 100–102). Increasing systemic circulation time of GO with high plasma concentration decreases reticuloendothelial system (RES) clearance and reduces drug accumulation in the normal organs to reduce toxicity and to enhance the EPR effect (103). Carmustine [1,3-bis(2-chloroethyl)-1-nitrosourea (BCNU)] is a chemotherapeutic drug commonly used to treat glioma. GO can be modified with polyacrylic acid (PAA) to improve its aqueous solubility and increase cell penetration efficacy. Lu et al. found that PAA–GO covalently combined with BCNU significantly prolonged the half–life of BCNU and effectively increased the intracellular drug concentration, thereby enhancing apoptosis of glioma cells *in vitro* (104). Wang et al. used PF127 to modify GO to enhance its water solubility and obtained PF127–GO–DOX, which significantly enhanced the tumor growth inhibition of glioma compared with pure DOX in glioblastoma U251 cell line (39). Lucanthone (Luc), APE-1 (Apurinic endonuclease-1) inhibitor, can reverse the glioma cell resistance to radiation and chemotherapy *in vivo* and *in vitro* (105–107). Sayan et al. coated GO with DSPE-PEG to load Luc, which significantly induced the cell death in glioblastoma U251 cell line (86). Modified GO can produce several killing effects against tumors, and the most common combination is chemo-photothermal therapy. Wang et al. designed a multifunctional platform based on GO, and the platform combined chemo-photothermal therapy, MRI targeting, and glioma targeting together, which provided a perspective way for glioma therapy *in vitro* and *in vivo* (108). Yang et al. modified GO with PEG and EGFR antibody to carry epirubicin (EPI), which significantly prolonged the survival of mice with glioma, and the treatment strategy included chemotherapy and photothermal therapy (109). Nucleolin is one of the major nuclear proteins, but it is overexpressed on the membranes of glioma cells and vascular endothelial cells, which makes it a promising target (110–113). AS1411 is a DNA aptamer rich in guanine, which can specifically bind to Nucleolin, and has good tumor targeting to anti-glioma (114). Du et al. coated GO with AS1411 to load B3, a berberine derivative, and proved the nanocarrier a promising treatment for tumors (115).

GO can also achieve targeted therapy of tumors through appropriate modification, including molecular targeting, external magnetic field targeting, and other targeting forms. Lactoferrin (Lf) is an iron-transporting serum glycoprotein that binds to receptors overexpressed on the surface of glioma cells and vascular endothelial cells of the BBB. Song et al. established the GO–Fe3O4–DOX delivery system and systematically evaluated its anti-glioma effect. The system was reported to prolong the action of DOX and increase uptake of the molecule by glioma cells, which displayed stronger cytotoxicity against C6 glioma cells (116). Angiopep-2 (ANG-2), a polypeptide and a specific ligand for the low-density lipoprotein receptor-related protein-1 (LRP-1), has a strong ability to pass through the BBB and has a high transcytosis capacity; it can act as a target to increase the ability to deliver drugs to the brain (117). Zhao et al. modified GO with ANG-2 and then attached DOX to the carrier system. The resulting nanocarriers more significantly enhanced tumor growth inhibition than DOX or DOX–GO both *in vitro* and *in vivo* (118). The glioma cell line U87–MG with high expression levels of EGFR on the surface is an optimized model for targeting by cetuximab (CET), an EGFR antibody. Lu et al. attached irinotecan (CPT-11) onto GO–CET and then added chitosan-g-poly(N-isopropylacrylamide) (CPN), a thermosensitive gel, and subsequently entrapped stomatin-like protein 2 (SLP2) short hairpin RNA (shRNA). The final GO–CET–CPT11–shRNA–CPN nanocarrier system targeted gliomas with a high expression level of EGFR, formed a hydrogel in the tumor area, prolonged the duration of action of CPT11, and induced cells apoptosis both *in vitro* and *in vivo* (119). This system was found to perform dual functions in chemotherapy and gene therapy, thus broadening the treatment options of glioma. The glioma surface is rich in folic acid (FA) receptors. Therefore, Wang et al. added FA and TMZ to GO and the system showed a pH-dependent response as well as sustained release properties after reaching the glioma area, effectively reducing the drug dose and prolonging its duration of action *in vitro* (120). Transferrin (Tf)-mediated transport has been proven to cross the blood-brain barrier, which makes Tf a potential molecular therapeutic target in the treatment in the treatment of glioma (121). In addition, Tf receptors are overexpressed on the surface of glioma cells (122). Hence, Liu et al. used Tf as a target functional group and DOX as a chemotherapeutic drug to construct a new type of glioma-targeted Tf–PEG–GO–DOX nanocarrier delivery system. *In vivo* experiments showed that the Tf–PEG–GO–DOX system effectively increased the intratumoral concentration of DOX and significantly prolonged the survival period of tumor-bearing rats, indicating that the system could effectively improve the efficacy of glioma treatment (123). CTX, a peptide of scorpion venom, has high selectivity for gliomas by binding to matrix metalloproteinase-2 (124). Consequently, Wang et al. modified GO with CTX and loaded DOX to form CTX–GO–DOX nanocarriers, which significantly increased the concentration of DOX in the tumor cells and ultimately enhanced the killing effect of the drug against C6 glioma cells *in vitro* (35). CTX–GO is, therefore, a promising drug delivery system for the targeting of gliomas. GO–Fe3O4 can be used for targeted delivery through a magnetic field; it can release the loaded drug in this field to induce the desired therapeutic effect. Wang et al. used GO–Fe3O4 to load TMZ and yielded the GO–Fe3O4–TMZ system, which was controlled by an external magnetic field and had a strong drug-loading capacity for TMZ with a strong killing effect on C6 glioma cells *in vitro* (125). External magnetic field targeting with GO can play a role not only in targeted drug delivery but also in auxiliary imaging. Sakine et al. modified GO with superparamagnetic iron oxide nanoparticles (SPION) and poly(lactic-co-glycolic acid) (PLGA) and then attached the radiosensitizing drug IUdR on it to form IUdR–GO–SPION–PLGA. The latter system was enriched in gliomas with the use of an external magnetic field during *in vivo* experiments and reported to significantly strengthen the apoptosis-inducing effect of IUdR during glioma radiotherapy when monitored under MRI (126, 127). This system can increase the therapeutic effect of IUdR, effectively reduce its dosage, and in turn reduce its toxicity to healthy biological tissues. There are a variety of other modified groups that are used to target gliomas, and the effectiveness of these groups is worthy of further exploration.

### 3.3 Immunotherapy of Graphene Oxide Nanocarriers

Tumor immunotherapy is mainly based on the relationship between the immune function of the body and the status of tumors, and it is important to find optimized intervention measures to regulate the body’s immune response to tumors to achieve anti-tumor effects (128). Dendritic cells (DCs) are the most important antigen-presenting cells known in current research to date. Under normal circumstances, there are very small amounts of DCs in the human body. Only once DCs can normally perform the antigen-presenting function can the body effectively recognize the pathogen, induce an immune response, and produce a normal immune response (129). DCs in cancer patients are defective and cannot present tumor antigens properly. Reactivating DCs to initiate anti-tumor immune responses has become an important hot spot in tumor immunotherapy in recent years. For central nervous system tumors, activated DCs can simultaneously promote the infiltration of lymphocytes in the tumor microenvironment and monitor the entire central nervous system accurately and specifically (130). GO excels in loading and delivering antigen (Ag). Wang et al. used the glioma peptide Ag from T98G, a human glioma cell line, to modify GO. GO–Ag has been reported to activate DCs *in vitro*, effectively inducing a specific anti-glioma immune response, promoting the arrival of lymphocytes and significantly upregulating the secretion of interferon-γ (IFN-γ) (131). GO–Ag provides a new idea for glioma immunotherapy; it is, therefore, important to find a safe form of Ag and also a safe form of the composite GO-Ag for use in clinical settings. Lu et al. developed Fe3O4 nanoparticles (FNPs)-rGO-PEG for used for MRI-guided photothermal-immunotherapy, which could activate DCs after laser irradiation *in vivo* (132). Indoleamine-2,3-dioxygenase (IDO) is an immunosuppressive enzyme capable of inhibiting T cells, and IDO inhibitors (IDOi) boost the efficiency of immune-based cancer therapies (133). Yang et al. established PEG-rGO-FA-IDOi, which could directly kill tumor cells under laser irradiation and active antitumor immune response *in vivo* (134).

### 3.4 Phototherapy of Graphene Oxide Nanocarriers

Phototherapy includes photothermal therapy (PTT) and photodynamic therapy (PDT). Photothermal therapy refers to injecting materials with strong light–to–heat conversion properties into the human body, targeting them to the tumor tissues, and irradiating them with a light source to kill tumor cells by converting light energy into heat (135, 136). GO is a potential photosensitizer. It can convert light energy into heat energy under 808 nm near-infrared laser irradiation, with most biological systems not being sensitive to light in this region. Therefore, the use of GO for photothermal treatment can kill tumor cells contactless and non-invasive. Furthermore, it is non-invasive and safer than conventional treatment methods such as surgery, chemotherapy, and radiotherapy (137, 138). Samira et al. found that IUdR–GO–SPION–PLGA has radiosensitizing and photothermal treatment effects on gliomas in the near–infrared (NIR)region, significantly increasing the glioma killing effect of IUdR *in vitro* (139). Li et al. coupled the targeting molecule Tf on the surface of GO to prepare functionalized GO–Tf–FITC microparticles, which were irradiated with infrared rays at 808 nm. Flow cytometry detected the killing effect on glioma U251 cells with GO–Tf–FITC, with the apoptosis and death indices of the GO–Tf–FITC group being significantly higher than those of the GO–FITC and blank control groups. Furthermore, functionalized GO–Tf–FITC were found to have a significant targeted photothermal killing effect on glioma U251 cells when viewed using fluorescence imaging following fluorescence labeling (140). Dong et al. further established GO–PEG–Tf as a drug nanocarrier and loaded it with DOX to perform dual functions in chemotherapy and photothermal therapy. The system showed a great killing effect on gliomas in *in vivo* and *in vitro* experiments (141). Omid et al. transformed rGO into reduced graphene oxide nanomeshs (rGONMs) using TiO_2_, and then used RGD to modify GO targeting of U87–MG to establish rGONM–PEG–Cy7–RGD (142). Owing to the strong NIR absorption ability of rGONMs, this system can significantly improve the absorption capacity of NIR, which enhances the photothermal therapy ability on gliomas. Joshua et al. also established a similar model and further proved that the system is efficient and could be used for effective photothermal therapy using a mouse glioma model with low-dose GO at a low laser power (143). The improvement of photothermal therapy capabilities with GO is also an important direction for future research.

Photodynamic therapy uses photo-biochemical processes to convert light energy into free radicals, which then play a role in inducing programmed cell death (136). U87–MG has been reported to cause an overexpression of integrin αvβ3 protein receptors (144). Pyropheophorbide-alpha (PPa) is a promising second-generation photosensitizer (145). Wei et al. prepared a GO carrier, PPa–GO–mAb, coupled with an integrin αvβ3 monoclonal antibody as the targeting ligand. The experimental results showed that this nanocarrier system could actively target U87–MG, through gathering around the mitochondria of glioma cells and producing free radicals to exert a killing effect under near–infrared laser irradiation. A CCK8 cytotoxicity test confirmed that the nanocarriers could effectively kill U87–MG cells, and the killing effect was concentration–dependent (146) (**Table 2**).

**Table 2:** A brief summary of GO in glioma therapeutic conditions.

Type of application	Graphene complex	Ref.
Direct Killing	GO	(94)
	GO/rGO	(88)
	rGO	(89)
	GO	(90)
	GO	(91)
	GO	(93)
	GO	(95)
	rGO	(96)
	rGO	(97)
	rGO-Arg	(98)
Drug Delivery	GO-γ-Fe_2_O_3_-CisPt	(147)
	Tf–PEG–GO–DOX	(123)
	GO-Fe_3_O_4_-Lf-DOX	(116)
	GO-PEG-DSPE-Luc	(86)
	GO-Gd-Let-7g-EPI	(148)
	PF127-GO-DOX	(39)
	PAA-GO-BCNU	(104)
	ANG-DOX-GO	(118)
	CPN-GO-CET/CPT11-shRNA	(119)
	GO-FA-TMZ	(120)
	GO-Fe₃O₄-TMZ	(125)
Immunotherapy	GO-Ag	(131)
	FNPs-rGO-PEG	(132)
	PEG-rGO-FA-IDOi	(134)
Phototherapy	rGO-PEG-RGD	(143)
	rGONM-PEG-Cy7-RGD	(142)
	GO-porphyrin-RGD	(149)
	PPa-GO-mAb	(146)
	IUdR–GO–SPION–PLGA	(139)
	GO-Tf-FITC	(140)
Drug Delivery and phototherapy	rGO-BSA-DOX	(138)
	rGO-AuNRVe-DOX	(150)
	MGMSPI-PEG-IP-DOX-Fe_3_O_4_	(108)
	PEG-NGO-C225/EPI	(109)
	AS1411-GO/B3	(115)
	GO–PEG–Tf-DOX	(141)

GO, Graphene oxide; rGO, reduced Graphene oxide; Arg, arginine; CisPt, Cisplatin; Tf, Transferrin; PEG, Polyethylene glycol; DOX, doxorubicin; Lf, Lactoferrin; DSPE, 1,2-Distearoyl-sn-glycero-3-phosphoethanolamine; Luc, Lucanthone; Gd, gadolinium; EPI, epirubicin; PF127, Pluronic F127; PAA, polyacrylic acid; BCNU, 1,3-bis(2-chloroethyl)-1-nitrosourea; ANG, Angiopep; CPN, chitosan-g-poly(N-isopropylacrylamide); CET, cetuximab; CPT11, irinotecan; shRNA, short hairpin RNA; FA, Folic acid; TMZ, Temozolomide; Ag, antigen; FNP Fe_3_O_4_, nanoparticle; IDOi, indoleamine-2,3-dioxygenase inhibitor; RGD, arginine–glycine–aspartic acid; rGONM, reduced graphene oxide nanomesh; Cy7, cyanine 7; PPa, Pyropheophorbide-alpha; mAb, monoclonal antibody; IUdR, 5-iodo-2-deoxyuridine; SPION superparamagnetic iron oxide nanoparticle; PLGA, poly(lactic-co-glycolic acid); FITC, Fluorescein isothiocyanate; BSA, bovine serum albumin; AuNRVe, ultrasmall plasmonic gold nanorod vesicle; MGMSPI, magnetic graphene-mesoporous silica; IP, interleukin-13-based peptide; NGO, nanographene oxide, C225, cetuximab.

## 4 Conclusions and Perspectives

In general, GO nanocarriers are widely studied in the treatment of gliomas because of their excellent physical and chemical properties and have shown broad application prospects in medicine, such as in chemotherapy, immunotherapy, and phototherapy (151, 152). Modified GO nanocarriers have shown long drug action time, good efficacy, low toxic and side effects, high drug loading, acceptable biosafety, easy to achieve controlled release of drugs, and low price, which make them promising drug carriers for clinical applications. Zhu et al. injected docetaxel-GO-CS gel into tumor tissues of mice *in vivo*, which gained a significantly longer drug action time (153). Pei et al. confirmed that pGO-Pt-DOX presented more therapeutic efficacy and less systemic toxicity than free drugs *in vitro* and *in vivo* studies (154). Islami et al. achieved controlled release of quercetin (Qu) with Qu-hyperbranched polyglycerol (HPG)-GO, and Qu-HPG-GO revealed significant improvement in drug loading (155). After further exploration, the potential of GO nanocarriers in the clinical applications of gliomas will be limitless.

At present, the use of GO as a nanocarrier is still in the preliminary stages of research; there are still many problems to be solved, such as the effectiveness and safety of functionally modified GO nanocarriers in the body and their distribution in tumor tissues. GO is more water-soluble than graphene, but it tends to aggregate under physiological conditions, even leading death to mice (156). The modification GO surface can purposefully change its physical and chemical properties and biocompatibility to improve the drug carrier efficiency (53). Covalent modification of GO often requires multi-step chemical reactions, which may affect the activity of biomolecules, while non-covalent modification can avoid this deficiency, which is only limited to chemical or biological molecules with a specific structure (157). There is still room for improvement in the preparation and modification of GOs, which is worthy of further optimization research. The relationship between GO functionalization and biological system and the mechanism of biological clearance is yet to be completely understood. Key theories for controlling drug release of GO nanocarrier are still immature. The current GO nanocarriers are mainly used for drug loading *via* non-covalent interactions, which makes the drug loading amount unstable, and the drug loading stability of the GO nanocarriers is even more debatable (158). GO could passively accumulate to glioma tissue due to the EPR effect (24). Besides passive targeting such as EPR, practicing active targeting will improve clinical practicability. Hence, its targeting still needs to be improved and optimized. In addition, multifunctional and multi–target modifications of GO nanocarriers to improve the specificity of targeting gliomas and prolonging the retention time in tumors should also be the focus of future research, such as a combination of chemotherapy, immunotherapy, and phototherapy (152). After the release of loaded drugs, the elimination of GO in the body is also an issue that must be paid attention to in cancer therapy (159). GO is mainly used to load small molecule drugs, but not biomacromolecules such as DNA and protein, and a number of studies on GO are carried out *in vitro* for the potentially harmful effects, lacking *in vivo* experimental data (119, 160). At present, many studies of GO nanocarriers focus on classical chemotherapy drugs, anti-tumor traditional Chinese medicine components, and gene drugs. Further research is needed to improve the diversity of loaded substances and explore *in vivo* processes (161). In conclusion, there are still many problems in the study of GO, which requires scientific research teams to strengthen disciplinary integration and jointly solve the difficulties.

## Author Contributions

BW carried out the primary literature search, drafted and revised the manuscript, and participated in discussions. HG, HY, YC, HX, and GZ helped modify the manuscript. All authors read and approved the final manuscript.

## Funding

We acknowledge the funding support from National Nature and Science Foundation of China (Grant Number: 81772684), the S&T Development Planning Program of Jilin Province (Grant Number: 20200201469JC, 20200201613JC, 20200404101YY, and 20200201388JC), and Foundation from the Development and Reform Commission of Jilin Province (Grant Number: 2017C059-2, 2017C059-5, and Chinese People**’**s Brain Neural Network Research and Innovation Cooperation Platform Construction Project).

## Conflict of Interest

The authors declare that the research was conducted in the absence of any commercial or financial relationships that could be construed as a potential conflict of interest.

## Publisher’s Note

All claims expressed in this article are solely those of the authors and do not necessarily represent those of their affiliated organizations, or those of the publisher, the editors and the reviewers. Any product that may be evaluated in this article, or claim that may be made by its manufacturer, is not guaranteed or endorsed by the publisher.
